# The impact of leader safety communication on work engagement under pandemic: The effect of OBSE and anxiety based on COVID-19

**DOI:** 10.3389/fpubh.2023.1082764

**Published:** 2023-02-07

**Authors:** Xingchi Zhou, Yujie Guo, Yuhao Liu

**Affiliations:** School of Management, Wuhan Textile University, Wuhan, China

**Keywords:** JD-R model, safety communication, work engagement, organization-based self-esteem, anxiety, COVID-19

## Abstract

**Introduction:**

The outbreak of COVID-19 has a great impact on employees daily work and psychology. Therefore, as leaders in organization, how to alleviate and avoid the negative impact of COVID-19 so that employees can maintain a positive working attitude has become a problem to be worthy paying attention.

**Methods:**

In this paper, we adopted a time-lagged cross-sectional design to test our research model empirically. The data from a sample of 264 participants in China were collected using existing scales in recent studies, and were used for testing our hypothesizes.

**Results:**

The results show that leader safety communication based on COVID-19 will positively affect employees' work engagement (b = 0.47, *p* < 0.001), and organization-based self-esteem plays a full mediating role in the relationship between leader safety communication based on COVID-19 and work engagement (0.29, *p* < 0.001). In addition, anxiety based on COVID-19 positively moderates the relationship between leader safety communication based on COVID-19 and organization-based self-esteem (b = 0.18, *p* < 0.01), that is, when anxiety based on COVID-19 is at higher level, the positive relationship between leader safety communication based on COVID-19 and organizational-based self-esteem is stronger, and vice versa. It also moderates the mediating effect of organization-based self-esteem on the relationship between leader safety communication based on COVID-19 and work engagement as well (b = 0.24, 95% CI = [0.06, 0.40]).

**Discussion:**

Based on Job Demands-Resources (JD-R) model, this paper investigates the relationship between leader safety communication based on COVID-19 and work engagement, and examines the mediating role of organization-based self-esteem and the moderating role of anxiety based on COVID-19.

## 1. Introduction

After a long period of confusion, isolation, anxiety and pain caused by COVID-19, employees may find it difficult to stay focused and engaged at work, which may affect their work behavior and performance (1). At the same time, employees' requirements for the safety and health of their working environment are also increasing. In recent years, more and more attention has been paid to workplace and occupational safety issues, especially the research on its antecedent factors is increasing gradually. Among them, many studies regard leadership as an important factor affecting organizational security, especially the influence of leadership behavior has attracted more attention. For example, some scholars have found that open and frequent communication

and interaction between leaders and subordinates is conducive to improving organizational safety and reducing accident rate (2) and some scholars also proposed that leaders' behaviors of information sharing and communication are one of the important factors affecting work and occupational safety (3). Leader communication is a bridge to convey behavior intention to employees, which can improve employee's identification with the organization (4).

In the field of safety management, some studies began to focus on safety communication (5). Some studies have further suggested that an important basis for judging whether an organization has high safety performance is whether there is open and fixed communication between leaders and subordinates in terms of safety (6). Many studies have found that safety communication is significantly related to safety performance indicators such as safety climate, culture and management safety commitment (2, 7, 8). Cigularov (9) stated that in a dynamic and rapidly changing work environment, more effective safety communication can reduce the possibility of employees being hurt. This shows the importance of safety communication in the workplace. In the context of COVID-19, one of the biggest challenges for enterprises in safety production and management is how to keep employees motivated. Improper safety management will cause depression and physical, mental or emotional disorders, which will affect the working state of employees and then affect the performance of enterprises. At the same time, effective safety management will make the enterprise form a good working atmosphere, and the employees will help each other to bring about the improvement of performance.

**Figure 1:**
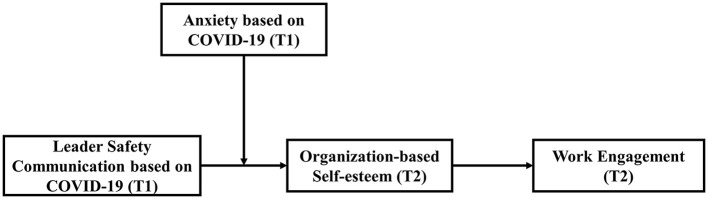
Proposed theoretical model.

Based on the JD-R theory, this study discussed the impact of leader safety communication based on COVID-19 as a job resource on employee's work engagement in the context of COVID-19 crisis in China, and also explored the mediating mechanism and boundary moderating mechanism between the two. The main theoretical contributions of this study are as follows: (i) the conclusion of this study enriches the research on the relationship between safety communication and its outcome variables and the related mechanism; (ii) it provides a new perspective for promoting employee's work engagement; (iii) in the context of COVID-19, the research path of JD-R theory has been expanded. The theoretical model of this study is shown in Figure 1 as follow.

The main aim of this study is to examine the relationship between leader's safety communication based on COVID-19 and employee's work engagement among Chinese workers under the pandemic, so as to further explore the underlying mechanism. We propose our hypotheses as follows:

H1: Leader safety communication based on COVID-19 has a positive impact on employee's work engagement.
H2: Leader safety communication based on COVID-19 has a positive effect on organization-based self-esteem.
H3: Organization-based self-esteem mediates the relationship between safety communication based on COVID-19 and employee's work engagement.
H4: Anxiety based on COVID-19 positively moderates the relationship between leader safety communication based on COVID-19 and organization-based self-esteem, that is, the higher the level of anxiety based on COVID-19, the greater the impact of leader safety communication based on COVID-19 on employee's organization-based self-esteem; The lower the level of anxiety based on COVID-19, the less the influence of leader safety communication based on COVID-19 on employee's organization-based self-esteem.
H5: Anxiety based on COVID-19 positively moderates the mediating role of organization-based self-esteem between leader safety communication based on COVID-19 and work engagement. The more anxiety based on COVID-19, the greater mediating role of organization-based self-esteem between leader safety communication based on COVID-19 and work engagement.

## 2. Literature review and hypotheses

### 2.1. Leader safety communication based on COVID-19 and work engagement

Some scholars believe that effective safety communication between leaders and subordinates is a two-way process involving information exchange (10), that is, leaders and subordinates, respectively, provide their own safety information. To be specific, the communication process can be divided into two aspects: on the one hand, the leader constantly gives safety information and relevant feedback to the subordinates, so that the subordinates can better understand the safety issues they need to face, such as the daily safety work procedures and compliance with the safety rules and regulations formulated by the organization (11); On the other hand, the discovery and concern of safety issues raised by employees can help leaders identify threats in the work environment before accidents occur and take control measures in advance (12). Moreover, the latter belongs to upward safety communication (that is, subordinates communicate safely with their leaders). If they feel comfortable in upward safety communication, such communication will increase the subordinates' sense of trust in the organization, thus further improving their motivation to maintain the working environment (13). Paixão et al. (14) reviewed publications related to leadership and healthcare published during COVID-19 crisis in 2020, and found that communication is one major and critical leadership feature that would help overcoming the crisis. Mearns and Reader (15) also found that if employees can feel that health is valued by their leaders and can freely discuss health and safety issues with their leaders, they will be rewarded with safe citizenship behavior, such as caring for the safe behavior of their colleagues, correcting potential safety problems, and reporting hazards. Some scholars pointed out that a well-organized communication atmosphere has a positive impact on employee's work engagement (16), and leader safety communication is conducive to the formation of a communication atmosphere. From the perspective of social exchange, the positive safety communication between leaders and subordinates may indicate that leaders care about the safety and wellbeing of subordinates, thus prompting subordinates to take initiative and make greater efforts in return for leaders and organizations (11). At the same time, the communication between leaders and subordinates is a process to generate trust and credibility (17), which can promote employee's work engagement (18).

Work engagement refers to the positive attitude of employees who are persistent, voluntary and willing to devote themselves to their work, including three dimensions of vigor, dedication, and absorption (19). There is a wealth of research on work engagement, recently Mazzetti et al. (20) adopted meta-analysis to review on the antecedents and consequences. They categorized the antecedents of work engagement into five aspects and consider leadership as one important impact factor to work engagement (20). Other research also indicates that leadership style can improve employee's work engagement by giving them clear tasks and vision of goals, as well as timely encouragement and support (21). For the outcomes lead by work engagement, scholars found that employees with high work engagement not only do well with their job performance but also have more confidence in their capacity for work, more job commitment, higher levels of resilience and focus, greater health, and life satisfaction, as well as less psychological stress turnover (20, 22). As it is found that employee's work engagement has a positive impact on both individual and organizational performance (23). In terms of the interaction between superiors and subordinates, some scholars also found that the tacit understanding between superiors and subordinates can make employees clearly analyze the tasks and requirements arranged by leaders, reduce the errors caused by information understanding, complete tasks arranged by leaders on time and receive recognition, so as to promote work engagement (24).

JD-R model (25) proposed that job demands and job resources are two basic working conditions of organizations (26). Among them, job demands mainly focus on the aspects that consume individual vigor and energy, such as workload, complex tasks, emotional demand and conflicts; job resources are mainly focused on helping employees deal with job demands and achieve goals, such as performance feedback, social support, skill diversity and other incentive work characteristics (27). These characteristics satisfy the basic psychological needs of employees (competence, relationship and initiative) (28). Existing studies have shown that when job resources cannot meet job demands, it will produce energy consumption effect on employees through the stress process, and when job resources can meet job demands, it will produce positive work results through the incentive process (26). Because job resources provide the possibility for individuals to achieve their goals and meet people's basic needs, it has a positive incentive effect on work engagement (i.e., a state of energy, dedication, and concentration) (29). The outbreak of COVID-19, enterprises should take reasonable prevention and control measures, which will reduce their profits and even face the risk of closing down, for employees, the risk of layoffs, pay cuts, health problems and home office increases dramatically, resulting in tension and anxiety among employees (30). Facing these situations will reduce the work motivation of employees, thus affecting their work engagement. Wood (31) found the positive correlation between job anxiety and job demands. Thus, safety communication is particularly important in employee's daily work. Therefore, we define the safety communication of leader in the context of COVID-19 as the leader safety communication based on COVID-19, which can be used as a job resource to meet the psychological needs of employees at work and reduce their psychological pressure. For example, when the product sales decreased, the leader informed that the online sales model could increase the sales, which could relieve the anxiety of employees to a certain extent. Business cuts leave employees idle, and leaders shift their attention from anxiety by encouraging them to learn more skills on their own. When employees are depressed, leaders encourage them by asking and comforting them (30). Smith and Dyal (32) studied work engagement from the perspective of security and found that one of the important factors influencing job engagement is safety participation, and considered safety participation as a kind of safety behavior, which relies on open communication, cohesion, trust, respect, and shared information. It can be seen that safety communication will have a positive impact on work engagement. Based on JD-R theory, leader safety communication based on COVID-19 is a kind of leader safety communication behavior under the current epidemic situation, which can be regarded as a kind of job resource to alleviate the tension and anxiety of employees caused by COVID-19, meet the psychological work needs of employees through incentive path, keep them in a positive, relaxed and happy state, improve job satisfaction and then strengthen work engagement. To sum up, we propose:

Hypothesis 1: Leader safety communication based on COVID-19 has a positive impact on employee's work engagement.

### 2.2. Leader safety communication based on COVID-19 and organization-based self-esteem

Relevant studies suggested that self-esteem expresses people's positive or negative attitude toward themselves, and self-esteem is defined as the evaluation made by individuals to themselves (33–35). Self-esteem also indicates the degree to which an individual believes that he/she is capable and reflects his/her value judgment (36). Individual organization-based self-esteem (OBSE) is a concept derived from the perspective of organization, which is defined as the degree to an individual considers himself capable, meaningful and valuable as a member of an organization (36). Studies have shown that factors such as the high-quality relationship between employees and leaders and leaders' trust in employees can promote the formation of organization-based self-esteem of employees, and the communication between superiors and subordinates is the basis of the formation of high-quality relationship (37, 38). Some scholars argue that in the process of communication with leaders, employees can obtain and analyze the information conveyed by leaders and judge whether they are valued, so as to strengthen or weaken their initiative in work (39). Cropanzano and Mitchell (40) proposed that communication generated by work in an organization can signal to employees that they are valued by the organization, thus improving the level of organization-based self-esteem of employees. During the COVID-19 pandemic, employees are faced with the risk of being laid off. When considering employment issues, they will naturally have a sense of work insecurity, accompanied by feelings of insecurity and anxiety (41). Based on the JD-R theory, leader safe communication based on COVID-19 can be regarded as a job resource, alleviating the negative impact of such psychological needs on the health and wellbeing of employees (25), making employees directly feel valued and improving their organization-based self-esteem. To sum up, we propose:

Hypothesis 2: Leader safety communication based on COVID-19 has a positive effect on organization-based self-esteem.

### 2.3. The mediating role of organization-based self-esteem

Furthermore, organization-based self-esteem can effectively promote employee's work engagement. According to JD-R theory, organization-based self-esteem can also be used as a job resource to promote a variety of work-related outcomes (42). A higher level of environmental uncertainty will make employees think that the organization is at risk and produce tension and anxiety (43), which will have a negative impact on organization-based self-esteem (44). The concern, encouragement and support of organizations and leaders for employees have a positive impact on the improvement of organization-based self-esteem (45). Work stress also affects organization-based self-esteem. Some studies have found that work stress, such as role conflict, role ambiguity and role overload, has a negative effect on organization-based self-esteem (44). Meanwhile, organization-based self-esteem can effectively alleviate the negative impact of job insecurity on work engagement (46). Employees with high level organization-based self-esteem believe that they are trusted in the organization, valuable, and contribute to the organization (47). From the perspective of intrinsic motivation, when employees perceive that they are valued and useful in the organization, they will be more inclined to do more beneficial behaviors for colleagues and the organization at work, so as to help the organization achieve its goals (48). Employees with low level organization-based self-esteem are more likely to believe that they are not valued in the workplace, which will weaken employee's work motivation and work behavior (49). In addition, Hui and Lee (50) found in their study that, compared with employees with high levels of organization-based self-esteem, employees with low levels of organization-based self-esteem showed lower organizational commitment and higher absence rates, and were unwilling to engage in behaviors that beneficial to organization. A high level of organization-based self-esteem means a high level of self-perceived value. This psychological state satisfies and strengthens individual demands, thus making the organization a demand fulfiller in the life of employees (36). Therefore, employees with high level organization-based self-esteem may have more proactive behaviors at work, while employees with low level organization-based self-esteem may have less proactive behaviors at work. To sum up, we propose:

Hypothesis 3: Organization-based self-esteem mediates the relationship between safety communication based on COVID-19 and employee's work engagement.

### 2.4. The moderating effect of anxiety based on COVID-19

A phobia is a specific form of anxiety disorder, defined as a persistent and excessive fear of an object or situation, which can be divided into three categories: social phobia, public place phobia and specific phobia (51). Arpaci et al. (52) identified “COVID-19 phobia” as a fear of COVID-19 and classified it as one of the specific phobias in the DSM-V (Diagnostic and Statistical Manual of Mental Disorders, 5th Edition). According to the DSM-V criteria, the main characteristic of a particular phobia is a fear or anxiety that is limited by the source of the fear. COVID-19 causes anxiety in people that coexist with suicidal tendencies, depression, and physical, mental, or emotional disorders (53–56). At the same time, people's disproportionate cognitive, emotional or behavioral responses to objects and events related to the COVID-19 pandemic can also have serious negative physiological and psychological effects (52). Since the COVID-19 epidemic has seriously disrupted people's daily life, it will also cause panic and psychological anxiety (57–59). Previous studies have shown that natural disasters such as earthquakes and tsunamis; man-made disasters such as explosions, wars or terrorism; epidemics such as MERS, SARS or Ebola cause harmful emotions such as fear, anxiety, depression, hopelessness and hostility in the short and long term (60–63). COVID-19 is expected to cause more psychological anxiety problems due to easy transmission, lack of specific drugs and high virus mortality (57, 64, 65). Based on the above studies, this article referred to the anxiety caused by COVID-19 as the anxiety based on COVID-19. When psychological anxiety exceeds a certain level, employees will perform negative behavior. For example, Jones et al. (66) found that psychological anxiety would lead to the consequences of employee dimission, low attendance rate and low work performance. Other study showed that there is a significant negative correlation between job anxiety and job satisfaction. The positive correlation between job anxiety and absence has also been proved in the study (66). Among the three dimensions of work engagement, absorption means that employees have a highly focused working state and will not be easily disturbed by external factors; vigor refers to the staff have abundant energy into the work; dedication refers to the selfless attitude that employees have in their work (19). As a leader, it is important to detect the early symptoms of anxiety based on COVID-19 in employees and provide timely psychological support (57, 67). When employees have little anxiety based on COVID-19, their mental resilience can cope with the pressure brought by anxiety well, and at this time, the safety communication based on COVID-19 is of little effect. With the increase of employee's anxiety based on COVID-19, employee's psychological resilience is not enough to cope with the increased pressure. At this time, leader safety communication based on COVID-19 makes employees clearly feel the concern of the organization for their own health and safety, which can effectively alleviate the pressure and negative emotions brought about by the anxiety based on COVID-19, and improve the organization-based self-esteem of employees. To sum up, we propose:

Hypothesis 4: Anxiety based on COVID-19 positively moderates the relationship between leader safety communication based on COVID-19 and organization-based self-esteem, that is, the higher the level of anxiety based on COVID-19, the greater the impact of leader safety communication based on COVID-19 on employee's organization-based self-esteem; The lower the level of anxiety based on COVID-19, the less the influence of leader safety communication based on COVID-19 on employee's organization-based self-esteem.

In the above discussion, leader safety communication based on COVID-19 increased employee's work engagement through the mediating role of organization-based self-esteem. The effect of the safety communication based on COVID-19 on the organization-based self-esteem of employees will be different with the difference of the level of anxiety based on COVID-19, which will affect the work engagement of employees. In other words, the mediating effect of leader safety communication based on COVID-19 on work engagement through employee's organization-based self-esteem is affected by the level of anxiety based on COVID-19. When the level of anxiety based on COVID-19 is low, leader safety communication has only a small effect on the employee's work engagement through organization-based self-esteem, because the pressure generated by less anxiety is very small and controllable for the employee. For the employees with high level of anxiety based on COVID-19, they will face a lot of pressure and negative emotions, and the corresponding psychological demands will also increase. It is difficult to deal with them only by the employees themselves. At this time, the mediating effect of leader safety communication based on COVID-19 on improving work engagement through organization-based self-esteem is significantly enhanced. From the above inference, we can find that there is a complex relationship between leader safety communication based on COVID-19, anxiety based on COVID-19, organization-based self-esteem and work engagement. To sum up, we propose:

Hypothesis 5: Anxiety based on COVID-19 positively moderates the mediating role of organization-based self-esteem between leader safety communication based on COVID-19 and work engagement. The more anxiety based on COVID-19, the greater mediating role of organization-based self-esteem between leader safety communication based on COVID-19 and work engagement.

## 3. Methods

### 3.1. Study protocol

This study was approved by the Ethics Committee on Human Experimentation of Wuhan Textile University (reference number: 2020OB001). We promised that all questionnaire data will only be used for this study, and only researchers can access the data, and no other organization or individual can obtain the data.

### 3.2. Sample and procedures

This study adopts the questionnaire survey method and the data is collected in Wuhan in two waves with 1 month interval. We labeled each survey questionnaire with a unique ID in advance and made sure that the participants can not notice the ID easily, so as to enable anonymous completion of our questionnaires and reduce the social desirability bias. Then the questionnaires are distributed to enterprises located in Wuhan using snowball sampling method (68). The enterprises come from different industries including IT, education, finance, service, manufacturing, and the medical industry. Psychologically there is enough time and influence for coronavirus generating stress and anxiety on the workers in Wuhan city. For example, when delta variant outbreaks in August, 2021 in Wuhan, some areas organized COVID-19 nucleic acid PCR test for the residents more than 10 times within a month.

At time 1 of our data collection, we both mailed the first part of the questionnaires to our contacts in different enterprises in the first week of August, 2021. The contacts are voluntarily to participate in our survey and data collection, also they are informed in advance that the questionnaire was completed anonymously and confidentially, and they can quit if they don't feel like to participate. This is because the city is threatened by delta variant outbreak during the period which may trigger more psychological anxiety from people here. We collected demographic information and leader safety communication based on COVID-19. At time 2 of our data collection, we sent out the second part of the questionnaires to the participants who have completed the first stage survey. We collected the variables of employee's organization-based self-esteem, work engagement, and anxiety based on COVID-19 (T2). Finally, we managed to have 354 employees from 8 companies agreed to participate, and 284 fully completed two questionnaires of time 1 and 2. We set criteria to exclude invalid samples, such as questionnaires with incomplete demographic information, failed with bogus items, and filled with too much same score to each item, etc. After removing invalid samples, we got 264 valid samples to conduct analysis. Among them, 56.8% were male and 43.2% were female; 91.7% of them were under 40 years old; 63.3% of the employees have bachelor's degree; The working years were mainly 1–5 years, accounting for 34.5%, followed by 5–10 years, accounting for 33.7%.

### 3.3. Measurement scales and analysis tools

All the measurement scales were adapted from existing literature and gone through “translation and back-translation procedure” which was widely used in cross-cultural studies (69), so that all the scales can adapt to the Chinese language environments. All measures were rated on Likert five point scale where 1 = strongly disagree to 5 = strongly agree (see Appendix A for scale items). In this study, SPSS 22.0 was used to perform descriptive statistics and related analysis on the main variables, and Mplus 7.0 was used to analyze the validity factors to test the structural validity and distinguish the validity of the variables. In terms of hypothesis test, this study used SPSS 22.0 for multi-level regression analysis, and the bootstrapping analysis method was used to estimate the confidence interval of 95% of the effect value, so as to test the mediating effect and the moderated mediation effect.

#### 3.3.1. Leader safety communication based on COVID-19 (T1)

Adopting the leader safety communication scale compiled by Cigularov et al. (9), including 5 questions such as “I think my leader encourages everyone to communicate frankly and openly on safety issues.” Items are self-evaluated by employees in the first stage of data collection. The Cronbach's α coefficient of the scale in this study was 0.87.

#### 3.3.2. Organization-based self-esteem (T2)

A short version of the scale was used to measure the employee's organization-based self-esteem. The original scale was compiled by Pierce et al. (36) and consisted of 10 questions. In accordance with Gordon and Hood (70), this study selected three items with the highest factor load as the measurement scale, including “I am very trusted in the organization,” etc., which were self-evaluated by employees in the second stage of data collection. The Cronbach's α coefficient of the scale in this study was 0.85.

#### 3.3.3. Work engagement (T2)

We adopted the utrecht work engagement scale (UWES−9) compiled by Schaufeli et al. (19), including 9 items such as “I am immersed in work,” was self-evaluated by employees in the second stage of data collection. The Cronbach's α coefficient of the scale in this study was 0.95.

#### 3.3.4. Anxiety based on COVID-19 (T2)

The fear scale developed by Arpaci et al. (52) for COVID-19 was used to measure the negative effects of COVID-19 on individuals in psychological, physical, economic and social aspects. In this study, psychological dimensions were selected to measure the psychological anxiety of employees on COVID-19, including 6 questions such as “news of death related to COVID-19 makes me very anxious,” which were self-evaluated by employees in the first stage of data collection. The Cronbach's α coefficient of the scale in this study was 0.89.

#### 3.3.5. Control variables (T1)

Based on previous research experience on work engagement (71), this study took the employee's gender, age, education level and working tenure as the control variables for data collection and hypothesis testing, so as to exclude their influence on the research variables and ensure the accuracy of hypothesis testing.

## 4. Results

### 4.1. Confirmatory factor analyses, descriptive statistics, and correlations

Confirmatory factor analysis was first carried out on the 4-factor model, including the four variables of leader safety communication based on COVID-19, anxiety based on COVID-19, organization-based self-esteem and work engagement reported by employees. The CFA results were shown in Table 1. As can be seen from Table 1, the fitting indexes of the 4-factor model all meet the standard, χ^2^ = 355.88, df = 224, RMSEA = 0.06, CFI = 0.95, TLI = 0.94; fitting indexes of the 3-factor model does not meet the common standard (χ^2^ = 855.42, df = 227, RMSEA = 0.15, CFI = 0.73, TLI = 0.70), as well and 2-factor model (χ^2^ = 942.42, df = 229, RMSEA = 0.15, CFI = 0.70, TLI = 0.67) and 1-factor model (χ^2^ = 1178.15, df = 230, RMSEA = 0.18, CFI = 0.60, TLI = 0.56). Therefore, the 4-factor model has better fit than the other three competitive models, indicating that there is a certain degree of differentiation between the four variables. We conducted Harman single-factor test (37.08%) and the single factor CFA (χ^2^ = 1178.15, df = 230, RMSEA = 0.18, CFI = 0.60, TLI = 0.56), the results indicate that the common method deviation among the variables involved is not serious and within the acceptable range.

**Table 1:** Results of confirmatory factor analysis.

**Model**	**CMIN**	**DF**	**CFI**	**TLI**	**RMSEA**	**CMIN/DF**
Four-factor model: LSCBC, ABC OBSE, WE	**355.88**	**224**	**0.95**	**0.94**	**0.06**	**1.59**
Three-factor model: LSCBC + ABC, OBSE, WE	855.42	227	0.73	0.70	0.15	3.77
Two-factor model: LSCBC + ABC, OBSE + WE	942.42	229	0.70	0.67	0.15	4.12
One-factor model: LSCBC + ABC + OBSE + WE	1178.15	230	0.60	0.56	0.18	5.12

Values in bold indicate the best-fitting model. LSCBC, leader safety communication based on COVID-19; ABC, anxiety based on COVID-19; OBSE, organization-based self-esteem; WE, work engagement; CMIN, Chi-square; DF, Degree of Freedom; CFI, Comparative Fit Index; TLI, Tucker–Lewis Index; RMSEA, Root Mean Square Error of Approximation; CMIN/DF, Chi-square to DF Ratio.

Although this study adopts two stages to investigate the subjects and measures the variables involved at two time points, all measurement data are obtained by the same subject's self-assessment, which will inevitably lead to homology deviations. Harman single-factor test was used to verify the results. Four factors were separated out after non-rotating factor analysis for all the questions. The explanatory variation of the first factor was 37.08%, which did not exceed 40% of the recommended value. That is to say, there was no single factor to explain most of the variation, so the common method deviation of the collected data was not serious. In addition, the single factor CFA fitting indexes (χ^2^ = 1178.15, df = 230, RMSEA = 0.18, CFI = 0.60, TLI = 0.56) in Table 1 are very poor. It can be further seen that the common method deviation among the variables involved is not serious and within the acceptable range.

Descriptive statistics and correlation analysis results of each variable are shown in Table 2. As can be seen from Table 2, the correlation coefficient between leader safety communication based on COVID-19 and work engagement is 0.39, *p* < 0.01. The correlation coefficient between leader safety communication based on COVID-19 and organization-based self-esteem was 0.42, *p* < 0.01; The correlation coefficient between organization-based self-esteem and work engagement was 0.64, *p* < 0.01. The above results provide preliminary data support for subsequent tests.

**Table 2:** Descriptive statistics and correlations.

**Variable**	**Mean**	**SD**	**1**	**2**	**3**	**4**	**5**	**6**	**7**	**8**
1. Gender	0.56	0.49	1							
2. Age	2.48	0.67	−0.01	1						
3. Education	2.83	0.65	−0.04	0.05	1					
4. Tenue	2.92	1.06	0.09	0.77^**^	0.03	1				
5. Safety communication	4.04	0.71	−0.04	0.09	0.04	0.19^**^	1			
6. OBSE	3.46	0.86	0.02	0.13^*^	0.12	0.22^**^	0.42^**^	1		
7. Engagement	3.51	0.86	−0.08	0.18^**^	0.07	0.21^**^	0.39^**^	0.64^**^	1	
8. COVID-19 anxiety	3.19	0.97	0.06	−0.14^*^	−0.14^*^	−0.13^*^	0.03	−0.17^**^	−0.07	1

n = 264 employees.

* p < 0.05;

** p < 0.01. OBSE: organization-based self-esteem. Gender was coded as 1= male and 2= female. Age was coded as a four-level categorical variable: 1 = under 20, 2=20–30, 3=30-40 and 4 = over 40. Education was coded as a four-level categorical variable: 1 = high school or lower, 2 = 3-year collage, 3 = bachelor degree, 4= master degree and above. Working tenue was coded as a five-level categorical variable: 1 = < 1 year, 2 = 1–5 years, 3=5–10 years, 4= 10–15 years, and 5 = more than 15 years.

### 4.2. Hypothesis testing

In this paper, hierarchical regression method was used to preliminarily test the mediating and moderating effects, and then, according to Hayes' (72) research, we further verify the hypothesis of mediator variable and moderator variable with bootstrapping method. The regression analysis results are shown in Table 3. Model 1 in Table 3 examines the direct impact of leader safety communication based on COVID-19 on organization-based self-esteem of employees after controlling for the effects of gender, age, education level and years of working. In model 2, we controlled for the effects of gender, age, education level and years of working, and examined the effects of leader safety communication based on COVID-19 and anxiety based on COVID-19 interaction on organization-based self-esteem of employees. Model 3 was designed to examine the direct impact of leader safety communication based on COVID-19 on employee's work engagement after controlling the effects of employee's gender, age, education level and years of working. Model 4 was designed to examine the effect of organization-based self-esteem on work engagement after controlling for the effects of employee gender, age, education level, years of working and leader safety communication based on COVID-19.

**Table 3:** Hierarchical regression analyses.

**Dependent variable**	**OBSE**				**Engagement**			
	**Model 1**		**Model 2**		**Model 3**		**Model 4**	
	**b**	**SE**	**b**	**SE**	**b**	**SE**	**b**	**SE**
Intercept	0.89^*^	0.43	2.96^***^	0.26	0.85^*^	0.40	0.29	0.36
**Controls**								
Gender	0.04	0.10	0.04	0.09	−0.12	0.11	−0.15	0.08
Age	−0.07	0.11	−0.10	0.10	0.14	0.12	0.19	0.11
Education	0.14	0.07	0.09	0.07	0.06	0.08	−0.02	0.07
Tenue	0.15^*^	0.08	0.15^*^	0.07	0.08	0.07	−0.01	0.06
**Independent variable**								
Safety communication	0.46^***^	0.08	0.44^***^	0.06	0.47^***^	0.07	0.18^**^	0.07
**Mediator**								
OBSE							0.62^***^	0.05
**Moderator**								
COVID-19 anxiety			−0.14^**^	0.05				
**Interaction**								
Safety communication × COVID-19 anxiety			0.18^**^	0.07				
R-sq	0.21		0.27		0.20		0.46	

* p < 0.05;

** p < 0.01;

*** p < 0.001. OBSE, organization-based self-esteem.

According to the model 3 in Table 3, after excluding the influence of control variables, leader safety communication based on COVID-19 has a significant positive impact on work engagement, b = 0.47, *p* < 0.001, H1 is verified. Model 1 shows that after excluding the influence of control variables, leader safety communication based on COVID-19 has a significant positive impact on organization-based self-esteem, b = 0.46, *p* < 0.001, H2 is verified. Model 4 shows that after excluding the influence of control variables and leader safety communication based on COVID-19, organization-based self-esteem has a significant positive impact on work engagement, b = 0.62, *p* < 0.001. According to the results of mediating effect analysis, the mediating effect value of organization-based self-esteem between leader safety communication based on COVID-19 and work engagement is 0.29, *p* < 0.001, 95% confidence interval is [0.19, 0.41], excluding 0. In conclusion, the results show that organization-based self-esteem plays a mediating role between leader safety communication based on COVID-19 and work engagement, and H3 is verified.

**Figure 2:**
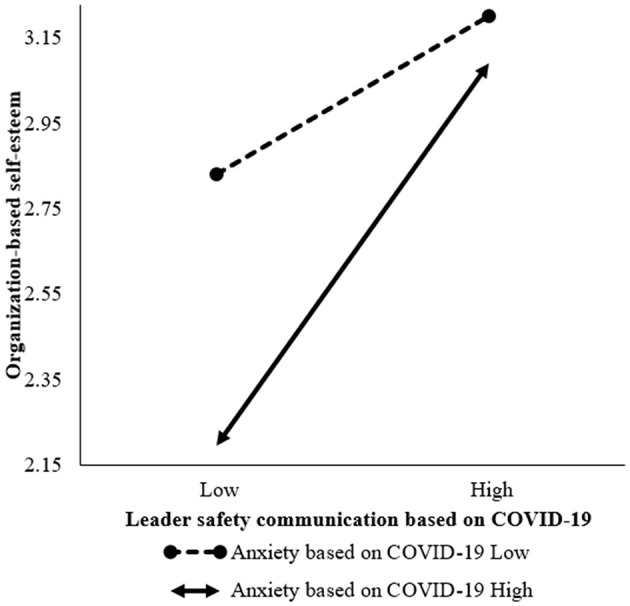
Interaction effect of leader safety communication based on COVID-19 and anxiety based on COVID-19 on OBSE.

In order to test the moderating effect of anxiety based on COVID-19 on the relationship between leader safety communication based on COVID-19 and organization-based self-esteem, we first centralize the variable data, and then use hierarchical regression method to test. Results as shown in model 2 in Table 3, after controlling for the main effect, the interaction item of leader safety communication based on COVID-19 and anxiety based on COVID-19 had a significant impact on organization-based self-esteem, b = 0.18, *p* < 0.01, indicating that anxiety based on COVID-19 has a moderating effect on the relationship between leader safety communication based on COVID-19 and organization-based self-esteem. In order to make a more intuitive observation on the moderating effect of anxiety based on COVID-19, according to the suggestion of Aiken et al. (73), the moderating effect diagram of leader safety communication based on COVID-19 and organization-based self-esteem on anxiety based on COVID-19 is drawn at the level of one standard deviation higher or lower than the average. The results are shown in Figure 2. The results showed that when the level of anxiety based on COVID-19 is low, the influence of leader safety communication based on COVID-19 on organization-based self-esteem is significant (b = 0.26, *p* < 0.01). When the level of anxiety based on COVID-19 is high, the influence of leader safety communication based on COVID-19 on organization-based self-esteem is stronger, b = 0.62, *p* < 0.001. Therefore, H4 is verified.

It can be seen from Table 4, when the level of anxiety based on COVID-19 is high, the indirect effect of leader safety communication based on COVID-19 on work engagement through organization-based self-esteem is stronger, with an effect value of 0.41, and the 95% confidence interval is [0.29,0.53] excluding 0. At a low level of anxiety based on COVID-19, the indirect effect value of leader safety communication based on COVID-19 on work engagement through organization-based self-esteem is 0.17, and the 95% confidence interval is [0.05,0.31], excluding 0. The difference of indirect effect values between the two groups above is 0.24, and the 95% confidence interval is [0.06, 0.40], excluding 0, indicating that the influence effect of the high-low group is significantly different. Therefore, H5 is verified.

**Table 4:** Mediating effect of organizational-based self-esteem on different levels of anxiety based on COVID-19.

**Moderator**	**Indirect effect**		**95%CI**	
**ABC**	**b**	**SE**	**Low**	**High**
High	0.41	0.06	0.29	0.53
Low	0.17	0.07	0.05	0.31
High-low group difference	0.24	0.09	0.06	0.40

ABC, anxiety based on COVID-19.

## 5. Discussion

On the basis of previous studies on safety communication, this study creatively combines the JD-R model to prove the influence of leader safety communication based on COVID-19 as a job resource on employee's psychology and behavior, and opens the “black box” of the influence of leader safety communication based on COVID-19 on employees' work engagement. The results show that: (i) leader safety communication based on COVID-19 can be regarded as a resource in work, which has a positive impact on employees' work engagement. Studies have shown that strengthening communication between leaders and subordinates can more clearly convey leaders' expectations and value orientation, give employees a sense of psychological security and achieve higher organizational performance by strengthen their work motivation (74). Mazzetti et al.' (75) also provides empirical evidences that perception of a safety climate is associated with higher risk perception and safety knowledge, which in turn, results in a higher implementation of safety behavior. Thus, as “shared climate perceptions evolve as a result of ongoing member-leader and member-member interactions” (75), safety-conscious leaders can promote a safety climate within the workplace through communication and other means such as role modeling in the interpersonal interactions. In the context of the COVID-19 pandemic, employees are facing both the stress of their work commitments and the stress caused by the virus. At this time, leaders' communication about employees' physical and mental health can make employees feel that the organization is not only concerned about work performance, but also attaches importance to their health and safety (76), which can enhance employees' sense of belonging to the organization and are willing to devote more energy to their work; (ii) Organization-based self-esteem mediates the relationship between leader safety communication based on COVID-19 and work engagement, that is, leader safety communication based on COVID-19 affects work engagement through organization-based self-esteem. This is in line with the perspective of internal motivation. The safe communication of leaders makes employees feel that they are valued and useful in the organization, and then tend to do more beneficial behaviors at work (48); (iii) anxiety based on COVID-19 has a consistent positive moderating effect on the relationship between safety communication based on COVID-19 and work engagement, that is, compared with the employees with low anxiety based on COVID-19, when anxiety based on COVID-19 is in high level, the psychological anxiety based on the new coronavirus is higher, the positive relationship between leader safety communication based on COVID-19 and organization-based self-esteem and work engagement will be strengthened. Anxiety can lead to turnover tendency and work slack (66). It is crucial for leaders to provide timely psychological support to employees through communication (57, 67). The findings of this study are of great significance to the applied psychology research and practice of COVID-19. But also, we need to be careful with our results generalization, because at the time period from July to September, 2021, there may be a fatigue scenario exist due to the COVID-19 lock down and social restrictions. This may affect participants' work engagement as well as anxiety toward COVID-19 exogenously and naturally. But the effect was small, and future research could improve the problem.

### 5.1. Theoretical contribution

This study enriches the application of the JD-R model in the field of organizational behavior. For the first time, it considers leader safety communication under the background of COVID-19, and proves the importance of such safety communication in the workplace. At the same time, combining with JD-R model, this paper considers leader safety communication based on COVID-19 as a kind of job resource, and explores the positive effects of leader giving this kind of job resource on organization-based self-esteem and work engagement of employees. In the past, some scholars have studied humble leadership behavior (77) as an antecedent variable to affect organization-based self-esteem and work engagement, but there is no research on safety communication as a antecedent variable of this path, this study demonstrates the feasibility of this path. In addition, we also explored the moderating effect of anxiety based on COVID-19 on leader safety communication based on COVID-19 and organization-based self-esteem, and studied how anxiety based on COVID-19 moderates the mediating effect of organization-based self-esteem on leader safety communication based on COVID-19 and work engagement. This is more comprehensive and systematic than the study of a single mediating or moderating effect.

### 5.2. Practical implication

This study deepens the understanding of safety communication from the perspective of JD-R, and find a new path for management practice to improve employee's work engagement. In the last few years, valuable research on the protective role of leadership and communication on safety issue in the workplace have been conducted across several cultural contexts, suggesting that “compassionate, open, and highly communicative leaders foster a sense of purpose that can act to strengthen a unified public health approach” (78), and honest communication is critical (79). (i) due to the impact of COVID-19, employees are facing a sharp increase in risks such as unemployment, health and work safety. As a leader, it is necessary to find out the psychological changes of employees in time and provide appropriate psychological support to alleviate the anxiety caused by the COVID-19. For example, asking employees about their health condition, reminding them to wear masks and getting vaccinated; (ii) leader safety communication can create an atmosphere in which employees can communicate with each other imperceptibly, which can not only reduce the psychological pressure brought by COVID-19, but also enhance their feelings with colleagues; (iii), the conclusion of this study shows that safety communication based on COVID-19 can make employees feel cared and valued by their leaders, thus improving work engagement, which is positively correlated with innovation and performance of enterprises, and employees who devote themselves to their work will bring more contributions to the enterprise.

### 5.3. Limitation and future directions

The shortcomings of this study are as follows: (i) The mechanism of safety communication on employee outcome variables may be diverse, and future research should attempt to elaborate the mechanism of safety communication and work engagement from multiple perspectives; (ii) The data in this paper are from leaders and employees, which can control the influence of common method bias on the research results to a certain extent. However, the relationship between leader safe communication based on COVID-19, organization-based self-esteem and work engagement is only discussed from the individual level. In the future, we can adopt cross-level research methods to make a more comprehensive and systematic study of this mechanism from the individual, team, organization and so on. In the research design, the use of cross-sectional design, to reveal the causal relationship between variables will have limitations, later scholars can be at different points in time to measure each variable at the same time, so that the relationship between variables can be further analyzed. (iii) The data of this study may have the issue of convenience sample. Besides, the COVID-19 pandemic scenario is quite different across different countries and cultures, and employee in China may have very singular experience and very different perceptions than other part of the world. Although we feel our research model based on JD-R theory is robust across countries, future studies still need to test the results in different cultures to generalize the research findings.

## 6. Conclusion

This study explored the impact of leader safety communication based on COVID-19 on employee work engagement. According to JD-R theory, we have introduced OBSE as an important mediator and anxiety based on COVID-19 as a moderator. The research results show that the mediating effect of leader safety communication based on COVID-19 through OBSE is positively related to work engagement, and the mediating effect is enhanced by anxiety based on COVID-19.

The contribution of this study is mainly reflected in three aspects. First, study whether leader safety communication based on COVID-19 will have a positive impact on work engagement, thus expanding relevant research. Second, we provide a theoretical basis for the mechanism of the relationship between leader communication and work engagement, and explore the boundary condition of this relationship. Third, we also provide ideas for extending the research related to JD-R model.

## Data availability statement

The raw data supporting the conclusions of this article will be made available by the authors, without undue reservation.

## Ethics statement

The studies involving human participants were reviewed and approved by Ethics Committee of the School of Management at Wuhan Textile University. The patients/participants provided their written informed consent to participate in this study.

## Author contributions

Conceptualization and investigation: XZ and YL. Methodology and project administration: YL and YG. Formal analysis: YL. Resources and writing—review and editing: XZ and YG. Writing—original draft preparation and funding acquisition: XZ. All authors contributed to the article and approved the submitted version.

## Appendix A

**Appendix A:** Scale items.

**Leader safety communication based on COVID-19 scale items (Cronbach's Alpha = 0.87)**
1. I feel comfortable discussing safety issues with my immediate.
2. I try to avoid talking about safety issues with my immediate foreman.
3. I feel that my immediate foreman openly accepts ideas for improving safety.
4. I am reluctant to discuss safety-related problems with my immediate foreman.
5. I feel that my immediate foreman encourages open communication about safety.
**Organization-based self-esteem scale items**
**(Cronbach's Alpha = 0.85)**
1. I count around here.
2. I am trusted around here.
3. There is faith in me around here.
**Work engagement scale items (Cronbach's Alpha = 0.95)**
1. At my work, I feel bursting with energy.
2. At my job, I feel strong and vigorous.
3. I am enthusiastic about my job.
4. My job inspires me.
5. When I get up in the morning, I feel like going to work.
6. I feel happy when I am working intensely.
7. I am proud of the work that I do.
8. I am immersed in my work.
9. I get carried away when I am working.
**Anxiety based on COVID-19 scale items**
**(Cronbach's Alpha = 0.89)**
1. The fear of coming down with coronavirus makes me very anxious.
2. I am extremely afraid that someone in my family might become infected by the coronavirus.
3. News about coronavirus-related deaths causes me great anxiety.
4. Uncertainties surrounding coronavirus cause me enormous anxiety.
5. The pace that coronavirus has spread causes me great panic.
6. I argue passionately (or want to argue) with people I consider to be behaving irresponsibly in the face of coronavirus.
